# Ketone body 3‐hydroxybutyrate mimics calorie restriction via the Nrf2 activator, fumarate, in the retina

**DOI:** 10.1111/acel.12699

**Published:** 2017-11-09

**Authors:** Yusuke Izuta, Toshihiro Imada, Ryuji Hisamura, Erina Oonishi, Shigeru Nakamura, Emi Inagaki, Masataka Ito, Tomoyoshi Soga, Kazuo Tsubota

**Affiliations:** ^1^ Department of Ophthalmology Keio University School of Medicine Tokyo Japan; ^2^ Department of Developmental Anatomy and Regenerative Biology National Defense Medical College Tokorozawa Japan; ^3^ Institute for Advanced Biosciences Keio University Tsuruoka Japan

**Keywords:** 3‐hydroxy butyrate, calorie restriction, ketone body, retinal protection

## Abstract

Calorie restriction (CR) being the most robust dietary intervention provides various health benefits. D‐3‐hydroxybutyrate (3HB), a major physiological ketone, has been proposed as an important endogenous molecule for CR. To investigate the role of 3HB in CR, we investigated potential shared mechanisms underlying increased retinal 3HB induced by CR and exogenously applied 3HB without CR to protect against ischemic retinal degeneration. The repeated elevation of retinal 3HB, with or without CR, suppressed retinal degeneration. Metabolomic analysis showed that the antioxidant pentose phosphate pathway and its limiting enzyme, glucose‐6‐phosphate dehydrogenase (G6PD), were concomitantly preserved. Importantly, the upregulation of nuclear factor erythroid 2 p45‐related factor 2 (Nrf2), a regulator of G6PD, and elevation of the tricarboxylic acid cycle's Nrf2 activator, fumarate, were also shared. Together, our findings suggest that CR provides retinal antioxidative defense by 3HB through the antioxidant Nrf2 pathway via modification of a tricarboxylic acid cycle intermediate during 3HB metabolism.

## 1 INTRODUCTION

Calorie restriction (CR) as the oldest and most robust dietary intervention provides many health benefits and has been used for health promotion among different races, religions, and regions worldwide (Morley, Chahla, & Alkaade, 2010). Although the relationship between CR and health benefits has been empirically demonstrated (Walford, Harris, & Gunion, 1992), the mechanisms underlying this process are multifactorial and still under intensive investigation. Complex association among nutrition‐sensing networks, mammalian target of rapamycin (mTOR; Sengupta, Peterson, Laplante, Oh, & Sabatini, 2010), AMP‐activated protein kinase (AMPK; Canto & Auwerx, 2011), peroxisome proliferator‐activated receptor co‐activator 1α (PGC‐1α; Finley et al., 2012), and sirtuin (Sirt) pathways (Imai, Armstrong, Kaeberlein, & Guarente, 2000) have recently been proposed as possible underlying mechanisms in rodents and nonrodent lower animals.

D‐3‐hydroxybutyrate (3HB), acetoacetate (AcAc), and acetone are physiological ketone bodies that are synthesized from fatty acids in the liver and serve as alternative energy sources during nutritional deprivation, including CR (ketogenesis; Krebs, 1961; Newman & Verdin, 2014). Among these, 3HB is an abundant ketone body circulating in the blood. Synthesized 3HB from ketogenesis is transported to peripheral tissues through the blood circulation, where it can be converted back to acetyl‐CoA and utilized in the TCA cycle as metabolic fuel (ketolysis). Recently, apart from its role as a metabolic fuel, the effects of 3HB associated with the benefits of CR were reported. The activity of a limiting enzyme of 3HB synthesis, mitochondrial hydroxymethylglutaryl‐CoA synthase (mHMG‐CoAS), is regulated through deacetylation by Sirt3 (Shimazu et al., 2010) and mTOR (Sengupta et al., 2010), master regulators of energy metabolic homeostasis signaling, and these activities have been linked to ameliorating diseases associated with oxidative stress. A more recent finding also showed that 3HB is an endogenous histone diacetyl‐lase inhibitor that helps cells resist oxidative stress (Shimazu et al., 2013). Furthermore, endogenous 3HB inhibits the activation of the NLRP3 inflammasome in macrophages, which is linked to chronic anti‐inflammatory effects (Youm et al., 2015). However, the overlapping mechanism between CR and 3HB has not yet been clarified.

The retina is a visual sensor localized in the posterior segment of the eye. It consists of various cell types: photoreceptor, horizontal, bipolar, amacrine, and retinal ganglion cells (RGCs). Major vessels of the retina are the central retinal blood artery and vein localized in the optic canal, and they spread from the inner side of the retina. Several studies have shown the beneficial effect of CR intervention for retinal degeneration in rodents. CR delays the age‐related degeneration of the retina, including RGC (Li, Sun, & Wang, 2003). CR attenuates the decrease in RGC activity caused by intraocular pressure elevation (Kong et al., 2012) and protects against ischemia/reperfusion stress‐induced RGC reduction (Kawai et al., 2001). A ketogenic diet and high‐fat/low‐carbohydrate and protein diet cause the elevation of endogenous 3HB and attenuate age‐related damage to RGC in rats (Zarnowski et al., 2015).

Here, we investigated the role of 3HB in the ameliorative effect of CR on retinal degeneration. Retinal degeneration was evaluated using an optic nerve and central retinal blood vessels transection (ONVT) model which simulates the acute retinal ischemic degeneration. Animals receiving CR were compared to animals receiving exogenous 3HB without CR. We focused on metabolic changes under these conditions. This study demonstrates not only the potential effect of CR on retinal degeneration, but also the novel role of 3HB in CR.

## 2 RESULTS

### 2.1 Intermittent fasting and exogenous, repetitive application of 3HB attenuate retinal degeneration

First, to verify the validity of the experimental conditions, changes in body weight, total calorie intake, serum biochemicals, and blood 3HB were compared between three groups:(i) allowed food and water ad libitum (AL), (ii) intermittent fasting (IF), and (iii) repeated administration of 3HB under the AL condition (3HB‐r).

**Figure 1:**
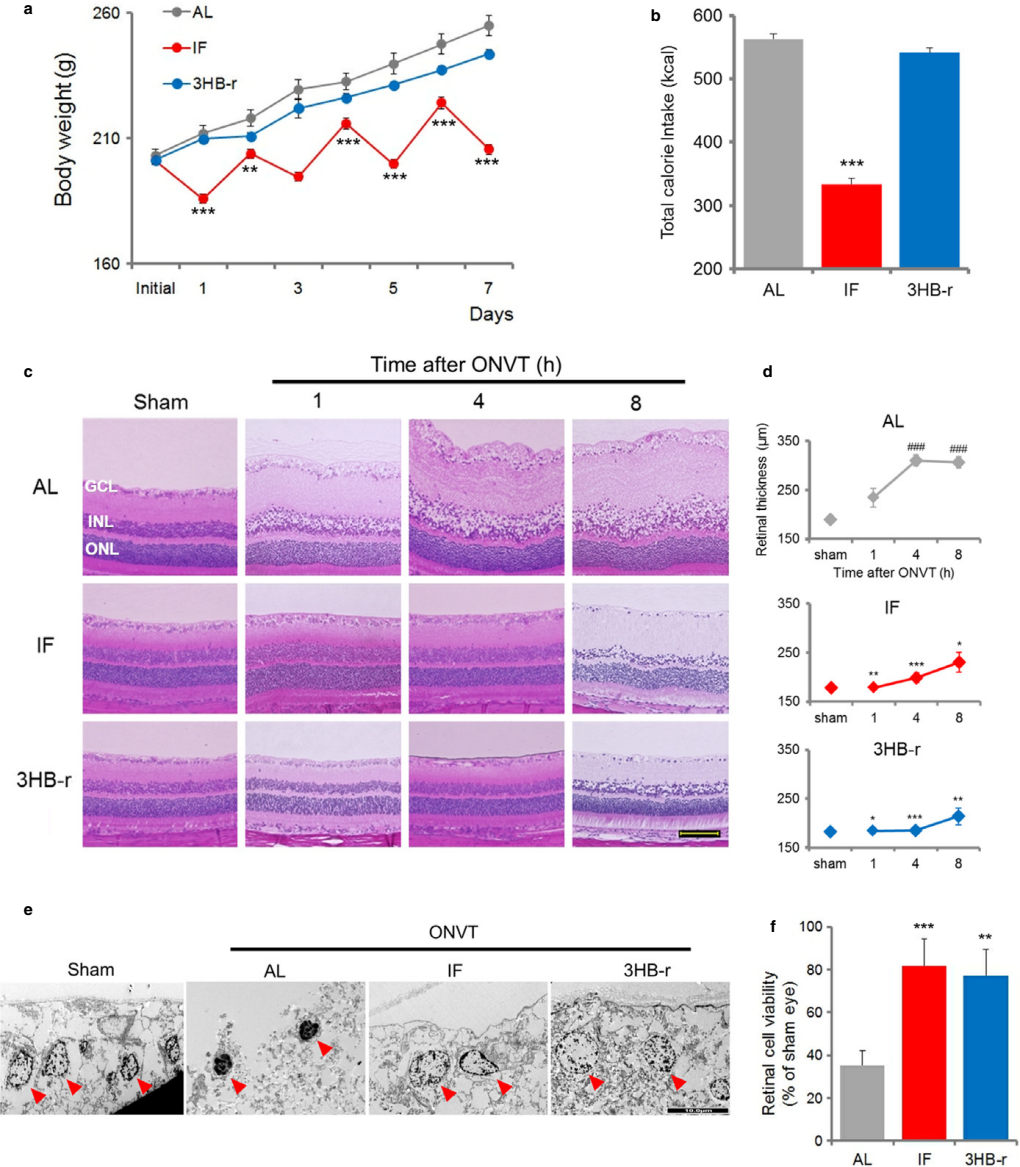
Intermittent fasting and repeated elevation of 3HB by exogenous administration of 3HB attenuate retinal degeneration induced by ONVT. (a) Body weight change (*n* = 6–7). (b) Total calorie intake (*n* = 5). (c) Temporal change in retinal segmental morphology. H&E staining of the retina was performed 1, 4, and 8 hr after ONVT. Scale bar, 100 μm. (d) Morphometric analysis of retinal degeneration. The analysis was performed at 1, 4, and 8 hr after ONVT (*n* = 6). (e) TEM analysis of ganglion cell layer 8 hr after ONVT. Arrow heads indicate nuclear. Scale bar, 10 μm. (f) Change in retinal viability. Viability was measured 4 hr after ONVT (*n* = 5–6). All data represent the mean ± *SE*, *p <* *.05, **p <* *.01, ***p <* *.001 vs. AL. ^###^ *p *<* *.001 vs. initial value (d)

Linear elevation of the body weight was observed in the AL group, and it increased by about 25% compared with the initial value on day 7 (Figure 1a). In the IF group, a decrease followed by an increase was observed repeatedly, corresponding to the daily fasting and feeding treatments, respectively. From days 2 to 7, the body weight significantly decreased to about 80%–90% compared with that in the AL group. In the 3HB‐r group, body weights were linearly elevated similar to that in the AL group. The total calorie intake in the IF group significantly decreased to approximately 60% of that of the AL group (Figure 1b). In the 3HB‐r group, the calorie intake was almost equivalent to that of the AL group.

Changes in the 3HB concentration in the vitreous fluid, forming a fluid border with the retina, were measured during IF and after 3HB subcutaneous administration (Fig. S1, and Data S1). 3HB increased gradually for 12 hr after food restriction, and then a marked rise was noted in the subsequent 12 hr. The concentrations of 3HB at 12 hr and 18–24 hr were four‐ and 10‐fold higher, respectively. The resumption of feeding markedly reduced 3HB to the same level as the initial value within 2 hr. The concentration and pattern of increase and decrease in response to fasting and subsequent feeding of the vitreous were the same as in the serum. 3HB subcutaneous administration resulted in an increase in the vitreous 3HB concentration, which peaked within 30 min, being 15‐fold higher than the initial value. Vitreous 3HB concentration then gradually decreased to the same level as the initial value within 3 hr (Fig. S1b). The concentration and pattern of increase and decrease in response to 3HB injection were the same in the vitreous fluid and serum. The peak 3HB concentration was similar to that observed in the IF group. Thus, the kinetics of the changes in 3HB concentration were suitable to simulate IF conditions.

Serum biochemical analysis indicating changes in levels of glucose and lipid‐related factors is shown in Table S1. In the IF group, concentrations of glucose, total lipids (TL), and triglycerides (TG) decreased to 50%, 60%, and 20%, respectively, compared with those in the AL group. These changes may be due to the interruption of carbohydrate and lipid absorption by fasting. Nonesterified fatty acids (NEFAs) increased to 170% when compared with that in the AL group. This increase was a lipolytic response of adipose tissue to carbohydrate deprivation by fasting. Released NEFA is used as a source for ketogenesis through beta oxidation of fatty acid in hepatic mitochondria as an alternative energy supplementation. These glucose and lipid metabolism changes were consistent with a previous CR report (Cahill & Veech, 2003). In the 3HB‐r group, the glucose level was similar to that of the AL group. Concentrations of TL, TG, and NEFA decreased to 80%, 70%, and 80%, respectively. These decreases may be associated with the decrease in food intake in the 3HB‐r group, which was approximately 90% of that of the AL group (Table S2). Altogether, these results suggest that 3HB‐r, namely the elevation of 3HB without CR, minimally affected the metabolic state in relation to ketogenesis.

Temporal change in retinal segmental morphology after optic nerve and central retinal blood vessel transection (ONVT) is shown in Figure 1c. In the AL group, edematous thickening in the inner nuclear layer (INL) to the ganglion cell layer (GCL) accompanied by swelling of ganglion cells appeared 1 hr after ONVT (Figure 1c, upper). The area of thickening in INL to GCL and swelling of ganglion cells increased until 4 hr after ONVT, and remained elevated up to 8 hr after ONVT. No pathological change in retinal segmentation was noted in the sham‐operated eye. Four hours after ONVT, the area of swelling increased and was maintained for up to 8 hr after ONVT.

Transmission electron microscopy (TEM) observation of ganglion cells at 8 hr after ONVT revealed dense aggregation of chromatin with the nucleus shrinkage, a characteristic of cell death progression, was observed in the AL group (Figure 1e left middle). No pathological change in the nuclear morphology was noted in the sham‐operated eye (Figure 1e left). These pathological changes are characteristic findings of an ischemic retina (Da & Verkman, 2004).

In contrast, in the IF and 3HB‐r groups (Figure 1c middle and lower, respectively), each retinal segment was similar to that in the sham‐operated eye at 1 and 4 hr after ONVT. Eight hours after ONVT, the edematous thickening was observed in the GCL and INL, although the changes were apparently smaller than those in the AL group. TEM observation of ganglion cells showed that the nuclear status was similar to that in the sham‐operated eye (Figure 1e left, right middle, and right).

The change in retinal thickness assessed by morphometric analysis is shown in Figure 1d. In the AL group, retinal thickness was significantly increased at 1, 4, and 8 hr after ONVT compared to that in the sham‐operated eye (Figure 1d, upper). In the IF and 3HB‐r groups (Figure 1d middle and lower, respectively), no significant change was observed in retinal thickness when compared with the sham‐operated eye. For the IF and 3HB‐r groups, retinal thickness was significantly increased compared with that in the AL group at 4 and 8 hr after ONVT.

We also analyzed retinal cell viability in freshly isolated retina 4 hr after ONVT (Figure 1f). The viability decreased to approximately 35%, 80%, and 75% compared with that in the sham‐operated eye in the AL, IF, and 3HB‐r group, respectively. Significant preservation of retinal cell viability was observed in the IF and 3HB‐r groups compared to that in the AL group.

Induction of edematous thickening in INL to GCL, swelling of ganglion cells, and decrease in retinal viability in freshly isolated retina after ONVT were not suppressed by single fasting (SF) and 3HB‐s injection (Fig. S2a, b, and c).

These results using IF, 3HB‐r with SF, and 3HB‐s suggest that repeated, prominent elevation of 3HB plays a role in retinal protection by IF.

### 2.2 The pentose phosphate pathway is preserved by IF and repeated 3HB exposure after ONVT

**Figure 2:**
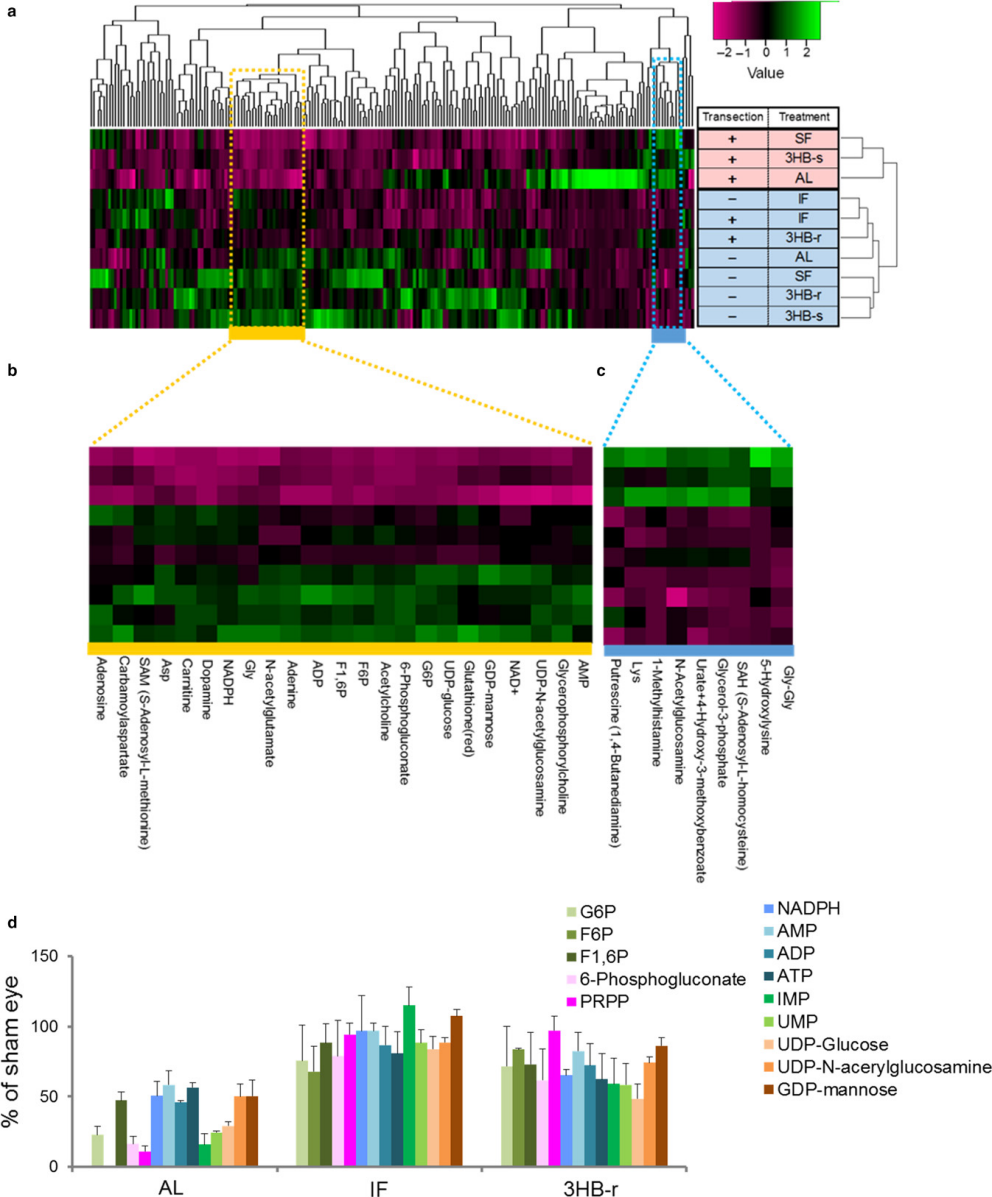
The pentose phosphate pathway is preserved by repeated elevation of 3HB induced by IF and 3HB‐r after ONVT. Retinas from 10 groups, AL, SF, IF, 3HB‐r, and 3HB‐s with or without transection, were used for analysis. (a) Hierarchical clustering heatmap of all 200 differentially expressed metabolites. Two clusters are differentially expressed between the nondegenerated retinas, IF and 3HB‐r with and without ONVT, and degenerated retinas in AL, SF, and 3HB‐s with ONVT. The yellow cluster represents 23 downregulated metabolites (b), and the blue cluster represents nine upregulated metabolites (c) in AL, SF, and 3HB‐s with transection, in which retinal degeneration was apparent after ONVT. (d) Comparison of changes in PPP‐related metabolites among IF, 3HB‐r, and AL groups. The ratio to the sham‐operated eye was calculated. Data represent mean ± *SE*,* n* = 3 measurements

Metabolomic analysis of the retina was conducted to identify the common metabolic pathways by which IF and 3HB‐r preserve retinal activity after ONVT. Isolated retinas from 10 groups treated with AL, SF, IF, 3HB‐s, or 3HB‐r, each with or without ONVT, were used for metabolomic analysis (Figure 2a). We focused on two clusters which were differentially expressed between nondegenerated retinas, IF, and 3HB‐r with ONVT and all treatments without ONVT, and degenerated retinas in AL, SF, and 3HB‐s with ONVT. In these clusters, 23 metabolites were downregulated (Figure 2b) and nine metabolites were upregulated (Figure 2c) in the degenerated retina. For the downregulated cluster, six metabolites were associated with the pentose phosphate pathway (PPP). The pentose phosphate pathway is a cytosolic process that is an alternative pathway for glucose oxidation. The role of PPP is to provide pentose phosphates for nucleotide synthesis and to generate reducing agents for many biosynthetic pathways and antioxidant defense (Eggleston & Krebs, 1974).

The changes in PPP and its downstream pathways as well as metabolites from metabolomic analysis in AL, IF, and 3HB‐r are summarized in Figure 2d. Glucose‐6‐phosphate (G6P), a starting metabolite of PPP, and the following PPP elements: fructose‐6‐phosphate (F6P), fructose‐1,6‐bisphosphate (F1,6P), and 6‐phosphogluconate, were identified in this cluster. NADPH is a cofactor involved in reductive biosynthesis generated from the first two steps of PPP. Reduced glutathione is generated at the expense of NADPH.

Pathways of purine and pyrimidine metabolism are connected downstream of PPP via the hub intermetabolite phosphoribosyl pyrophosphate (PRPP). Elements of the purine metabolism pathway, ADP, AMP, adenosine, and GDP‐mannose, and the pyrimidine metabolism pathway, UMP, UDP‐glucose, and UDP‐N‐acetylglucosamine, were also identified. Although PRPP does not belong to this cluster, it was upregulated in the IF and 3HB‐r groups compared with the AL group (Figure 2d, magenta bar). Certain metabolic pathways, other than PPP, were not revealed based on identified metabolites in these two clusters. In the SF and 3HB‐s groups, PPP and related metabolites decreased to about 50% or lower after ONVT as observed in the AL group (Fig. S2d). Overall, IF and 3HB‐r, but not SF and 3HB‐s, protected against retinal degeneration accompanied by PPP‐related metabolites due to the ischemic condition caused by ONVT, suggesting that the repeated elevation of 3HB during IF is critical for IF protective effects.

It is hypothesized that the retinal anti‐ischemic properties are established by repeated elevation of 3HB during IF, which causes the preservation of antioxidative PPP after ONVT. The role of 3HB as a metabolic fuel has been well recognized, although its involvement was excluded because SF and 3HB‐s had no protective effects.

### 2.3 PPP upregulation by elevation of 3HB suppresses ROS generation after ONVT

PPP generates NADPH for the detoxification of reactive oxygen species (ROS; Li et al., 2014). ROS generation from freshly isolated retinas and presence of an oxidative stress marker Nε‐(hexanoyl) lysine (HEL) were detected in retinal cross‐sections.

Reactive oxygen species generation 30 min after ONVT was approximately 1.5‐, 1.1‐, and 1.1‐fold compared with that in the sham‐operated eye in the AL, IF, and 3HB‐r groups, respectively. Significant suppression of ROS generation was observed in the IF and 3HB‐r groups compared to that in the AL group.

**Figure 3:**
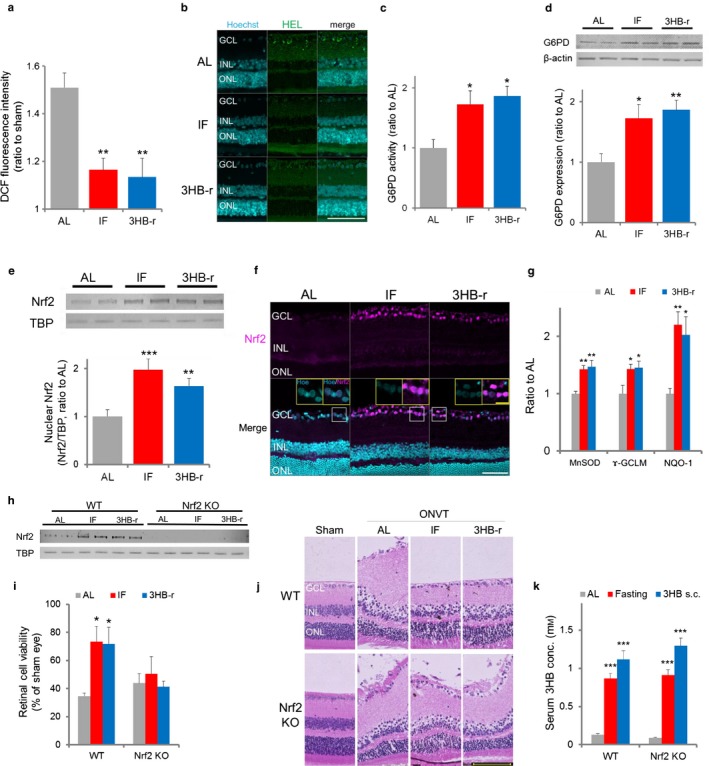
Repeated elevation of 3HB suppresses oxidative stress after ONVT via nuclear accumulation of antioxidative Nrf2 prior to ONVT. (a) ROS generation in the retina at 30 min after ONVT (*n* = 5). (b) Expression of retinal Nε‐(hexanoyl) lysine. Retinas excised 1 hr after ONVT were evaluated. Scale bar, 20 μm. (c) G6PD enzyme activity (*n* = 6–7). (d) G6PD expression level (*n* = 6). (e) Nrf2 nuclear accumulation levels. TBP was used as a loading control (*n* = 5). (f) Localization of Nrf2 in the retina. Scale bar, 100 μm. Insets show high‐magnification images. Scale bar, 20 μm (g) Expression levels of Nrf2 downstream factors, MnSOD, ɤ‐GCLM, and NQO1 (*n* = 5–6). Quantified images are shown in Fig. S3. (h) Nrf2 nuclear accumulation levels in Nrf2 knockout mice. Each lane represents retinal nucleus lysate from five mice of AL, IF and 3HB‐r. TBP was used as a loading control. (i) Changes in retinal cell viability in Nrf2 knockout mice. (j) Histopathological changes in the retina of Nrf2 knockout mice. (k) Changes in serum 3HB concentrations in Nrf2 knockout mice. Serum 3HB concentrations were measured 24 hr after fasting and 30 min after 3HB administration (*n* = 5–6). All data represent the mean ± *SE*. *p <* *.05, **p <* *.01, ***p <* *.001 vs. AL. Abbreviation used: Hoe, Hoechst 33342

In the AL group, HEL expression was observed predominantly in GCL and IPL after ONVT (Figure 3b, upper). In the IF and 3HB‐r groups, HEL was not detected in any of the layers. Consistent with metabolomic data, this finding suggested that the preserved retinal PPP presented ROS detoxification properties after ONVT.

### 2.4 Prior to ONVT, G6PD, a rate‐limiting enzyme of PPP as well as an upstream transcriptional factor of Nrf2, was upregulated by IF and 3HB‐r

As antioxidant PPP was preserved by IF and 3HB‐r, but not by SF or 3HB‐s, after ONVT, we hypothesized that the mechanisms for the preservation of PPP are established prior to ONVT. G6PD is a rate‐limiting enzyme of PPP which activates this pathway in response to the breakdown of NADPH (Ben‐Yoseph et al., 1996). In addition, G6PD is one of the biological protective factors orchestrated by Nrf2 (Mitsuishi et al., 2012), which is a transcriptional factor that ubiquitously responds to various physiological and pathological conditions.

The nuclear levels of Nrf2 and G6PD were evaluated prior to ONVT**.** In the IF and 3HB‐r groups, significant increases in G6PD activity (Figure 3c) and expression (Figure 3d), and nuclear levels of Nrf2 (Figure 3e) were observed compared with those in the AL group. Nrf2 localized in the nucleus of GCL cells (Figure 3f). This localization was consistent with previous reports (Fan, Xu, Jiang, & Qin, 2012; He et al., 2014). The number of Nrf2‐positive cells in GCL was higher in the IF and 3HB‐r groups than in the AL group.

Protein expression levels of downstream target genes of Nrf2 were confirmed by Western blotting. Significant increases in MnSOD (Lee et al., 2011), NQO‐1 (Venugopal & Jaiswal, 1996), and γ‐GCLM (Mulcahy, Wartman, Bailey, & Gipp, 1997) were observed in the IF and 3HB‐r groups compared to that in the AL group (Figure 3g and Fig. S3). In the SF and 3HB‐s groups, G6PD activity (Fig. S2e), Nrf2 level (Fig. S2f), and expression of the downstream targets of Nrf2 were similar to that in the AL group, consistent with retinal degeneration and metabolomic analysis data (Figs. S2g and S3). These results suggest that the repeated elevation of endogenous 3HB during IF activates G6PD by upregulating the Nrf2 pathway, which is associated with the preservation of PPP along with an antioxidative effect.

### 2.5 Nrf2 deletion abrogates the protective effect of IF and 3HB‐r on retinal degeneration induced by ONVT

To further confirm the above results, the effect of Nrf2 deletion on the protective effect of IF and 3HB‐r on retinal degeneration induced by ONVT was investigated by using an Nrf2 knockout mouse model. In wild‐type mice, similar to the result observed in rats, IF and 3HB‐r induced an increase in serum 3HB concentration (Figure 3k) and nuclear levels of Nrf2 (Figure 3h), ameliorating retinal degeneration (Figure 3j) and abrogating the decrease in viability induced by ONVT (Figure 3i). In Nrf2 knockout mice, although a significant increase in serum 3HB concentration was induced by IF and 3HB‐r, nuclear Nrf2 was not detected (Figure 3h). Retinal degeneration induced by ONVT was not suppressed by IF and 3HB‐r (Figure 3i). This result suggests that the suppression of retinal degeneration by IF or 3HB‐r is mediated through Nrf2.

### 2.6 Prior to ONVT, fumarate, an Nrf2 activator and a TCA cycle intermediate, was increased in the IF and 3HB‐r group

**Figure 4:**
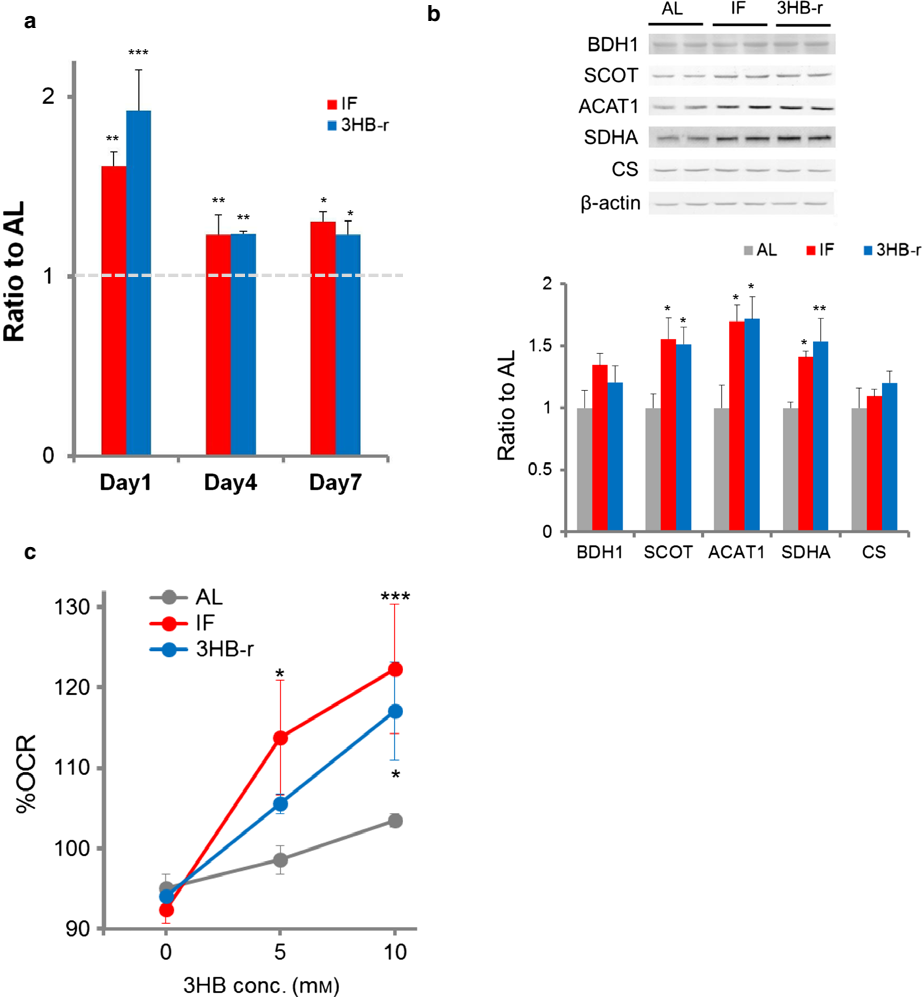
The Nrf2 activator, fumarate, is increased by repeated elevation of 3HB during IF prior to ONVT. (a) Changes in the fumarate level of the retina (IF: *n* = 5–6, 3HB: *n* = 4–5). (b) Expression levels of ketolysis and fumarate synthesis‐related enzymes, D‐3‐hydroxybutyrate dehydrogenase (BDH1), succinyl‐CoA‐3‐oxaloacid CoA transferase (SCOT), acetyl‐CoA acetyltransferase 1 (ACAT1), succinate dehydrogenase subunit A (SDHA), and citrate synthase (CS; *n* = 6). (c) Change in the oxygen consumption rate (OCR) induced by 3HB. Percentages to baseline value were calculated (*n* = 5–6). Data represent the mean ± *SE*, *p <* *.05, **p <* *.01, ***p <* *.001 vs. the AL value or initial value

A prominent supply of 3HB is expected to alter the balance of the pool of TCA cycle intermediates. Such intermediates serve as biosynthetic precursors as well as signaling messengers in cellular pathways (Czibik, Steeples, Yavari, & Ashrafian, 2014; Salminen, Kauppinen, Hiltunen, & Kaarniranta, 2014). Fumarate is an intermediate formed by the oxidation of succinate. Fumarate has a cytoprotective effect via activation of Nrf2 with the upregulation of protective antioxidant response element genes, by modification of the negative regulator KEAP 1 (Adam et al., 2011; Ashrafian et al., 2012). Temporal measurement of retinal fumarate showed that the content was significantly increased by IF and 3HB‐r compared with that in the AL group (Figure 4a).

To determine the metabolic pathway underlying ketone metabolism and increase in fumarate, the changes in expression level of enzymes involved in ketolysis and fumarate synthesis in the TCA cycle and TCA cycle flux rate were compared in the AL, IF, and 3HB‐r groups (Figure 4b). A significant increase in the level of succinyl‐CoA‐3‐oxaloacid CoA transferase (SCOT), a rate‐limiting enzyme of ketolysis that donates CoA to the TCA cycle during 3HB breakdown, was observed in the IF and 3HB‐r groups compared to that in the AL group. Levels of succinate dehydrogenase subunit A (SDHA), an enzyme that converts TCA cycle intermediate succinate to fumarate, and acetyl‐CoA acetyltransferase 1 (ACAT1), an enzyme that converts acetoacetyl‐CoA into two molecules of acetyl‐CoA, which are linked to SCOT's reaction, were also increased significantly (Figure 6). The levels of D‐3‐hydroxybutyrate dehydrogenase (BDH1), the first enzyme in the 3HB ketolysis pathway, and citrate synthase (CS), an enzyme synthesizing citric acid from acetyl‐CoA, remained unchanged among the groups.

The changes in retinal oxygen consumption rate (OCR), as an index of TCA cycle flux, in response to elevation of 3HB were evaluated in the freshly isolated retinal specimen from the AL, IF, and 3HB‐r groups. 3HB treatment increased OCR in a dose‐dependent manner in all groups. OCR levels were similar in the IF and 3HB‐r groups and significantly greater than that in the AL group (Figure 4c).

These findings suggest that Nrf2 is activated by an increase of the TCA cycle's Nrf2 activator, fumarate, through enhancing the ketolysis metabolic phase in the IF and 3HB‐r groups.

### 2.7 Aging suppresses the protective effect of IF and 3HB‐r on retinal degeneration by ONVT

Decline in energy expenditure is a characteristic of metabolic change in the aging process. Changes in the beneficial effect of CR (Li et al., 2003) or ketogenic diet (Zarnowski et al., 2015) have been reported based on the age at onset of these diets. In addition, ketone oxidation declines in an age‐dependent manner due to decrease in metabolic enzyme activity (Bregere, Rebrin, Gallaher, & Sohal, 2010). To investigate whether the age onset of CR alters its protective effect on the retina, the relationship between the protective effect of CR on the retinal degeneration induced by ONVT and elevation of serum 3HB was evaluated in the same experimental conditions using middle‐aged rats.

**Figure 5:**
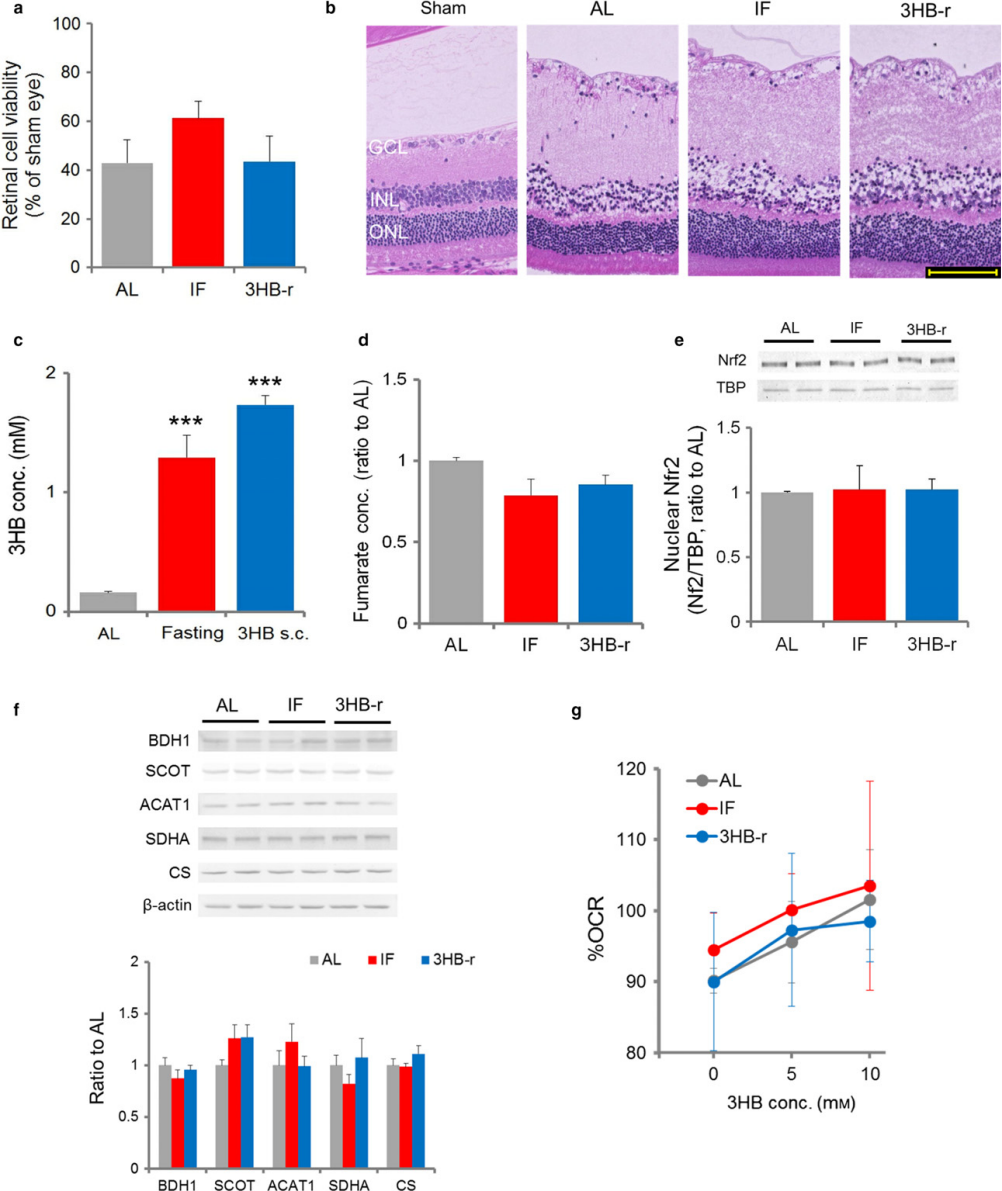
Aging suppresses the protective effect of IF and 3HB‐r on retinal degeneration induced by ONVT. (a) Change in retinal viability. Viability of freshly isolated retina from 4 hr after ONVT was measured (*n* = 4–5). (b) Pathological changes of the retina. Retinas from 4 hr after ONVT were evaluated. (c) Serum 3HB concentration. 3HB concentrations were measured at 24 hr after fasting, and 30 min after subcutaneous administration (1,000 mg/kg) of 3HB (*n* = 6). (d) Changes in the retinal fumarate level. (e) Nrf2 nuclear accumulation levels. (f) Expression levels of the retinal ketolysis and fumarate synthesis‐related enzymes, D‐3‐hydroxybutyrate dehydrogenase (BDH1), succinyl‐CoA‐3‐oxaloacid CoA transferase (SCOT), acetyl‐CoA acetyltransferase 1 (ACAT1), succinate dehydrogenase subunit A (SDHA), and citrate synthase (CS). (g) Change in the oxygen consumption rate (OCR) by 3HB (*n* = 5–6). Percentages to baseline value were calculated. Data represent the mean ± SE, *n* = 4 (d–f), ***p < .001 vs. the AL value.

IF and 3HB‐r did not preserve the induction of edematous thickening in GCL and INL and decrease in retinal viability in freshly isolated retina after ONVT when compared with AL (Figure 5a, b). Significant increase in serum 3HB concentrations, similar to that of the young rats, was observed in the IF and 3HB‐r groups compared with that in the AL group (Figure 5c).

No significant difference was noted in the level of BDH1, SCOT, ACAT1, SDH, and CS, TCA cycle's enzymes involved in ketolysis and fumarate synthesis, among the groups (Figure 5f). No difference was noted in the level of fumarate content (Figure 5d) and Nrf2 nuclear accumulation (Figure 5e). Although 3HB treatment increased OCR in a dose‐dependent manner in the freshly isolated retina in all groups, no significant difference was noted among the groups (Figure 5g).

Together with the results of young rats, these results suggest that impairment of ketolysis activity induced by aging abrogated the protective effect of IF and 3HB‐r on retinal degeneration induced by ONVT. Alternatively, these results provide additional evidence that enhancement of ketolysis plays a role in CR protective effects on the retinal degeneration after ONVT.

3HB is an endogenous HDAC inhibitor (Shimazu et al., 2013), and the inhibition of HDAC leads to the activation of the Nrf2 pathway (Wang et al., 2012), promoting protection against neuronal damage. Acetylation levels of histone H3, K9, K14, and K27 in the IF and 3HB groups were similar to those in the AL group (Fig. S4a). No change in downstream oxidative stress resistance factor, Foxo3a, and catalase (Shimazu et al., 2013) was observed among the groups (Fig. S4b). The discrepancy with previous data could be due to the variation of expression patterns of HDAC isoforms among tissues (Chausse, Vieira‐Lara, Sanchez, Medeiros, & Kowaltowski, 2015).

## 3 DISCUSSION

Ketone body metabolism is a metabolic pathway based on ketone body production with a release to the blood circulation mostly from the liver (ketogenesis) and utilization in extrahepatic tissues as an energy source (ketolysis). Investigation of the pleiotropic effects of 3HB on health benefits mainly focused on the ketogenesis pathway. In the present study, we demonstrate that upregulation of ketolysis in the retina, coupled with activated ketogenesis, plays a role in CR‐induced retinal antioxidative defense.

Ketogenesis is activated in concert with gluconeogenesis to provide an energy alternative to fatty acids during fasting (Garber, Menzel, Boden, & Owen, 1974). The prominent synthesis of 3HB through ketogenesis is one of the characteristic metabolic changes due to nutrient deprivation such as CR. In this state, the plasma 3HB level rises over four orders of magnitude, despite other substrates, free fatty acids, glycerol, and amino acids from gluconeogenesis being buffered within a far smaller range (Cahill, 1970). The urinary excretion level of 3HB is synchronized with that of plasma, suggesting that synthesis is excessive to deal with the cellular energy demand (Owen & Cahill, 1973) and it has been recognized as a waste material. However, little attention has been directed to clarify the relationship between the prominent synthesis of 3HB and health benefits.

In the present study, a repeated prominent increase of 3HB was accompanied by translocation of nuclear Nrf2 in the retina, which plays a pivotal role in the protective effect of IF on retinal degeneration after ischemia induced by ONVT. The mechanism underlying the translocation of nuclear Nrf2 by 3HB can be explained by elevation of retinal fumarate, an intermediate of the TCA cycle and an Nrf2 activator through the enhancement of ketolysis.

Ketolysis is a degradation process of 3HB into acetyl‐CoA through the following three phases (Krebs, 1970): (i) the oxidation of 3HB to acetoacetate coupling through NAD to NADH by the reversible enzyme BDH1; (ii) generation of acetoacetyl‐CoA from AcAc by CoA. This CoA is donated from the conversion of succinate‐CoA into succinate during the TCA cycle flux by SCOT; and (iii) acetoacetyl‐CoA is cleaved to generate two molecules of acetyl‐CoA by ACAT1, and it serves as a TCA cycle intermediate.

The TCA cycle is a central hub in the metabolism of fatty acids, amino acids, and carbohydrates initiated with the entry of acetyl‐CoA. The first half of the reaction of this cycle, the synthesis phase of the high‐energy intermetabolite succinyl‐CoA, is regulated allosterically in response to the energy redox state. This phase is initiated from the conversion of oxaloacetate with acetyl‐CoA into citrate. The second half of the pathway is the reproduction of oxaloacetate from succinyl‐CoA, along with the release of energy. The initial process of this phase, conversion from succinyl‐CoA to succinate, is concomitant with the second phase of ketolysis (Krebs, 1970). We found that 3HB treatment with or without CR elevated retinal fumarate accompanied by TCA cycle`s enzymes involved in ketolysis and fumarate synthesis, being independent of changes in the energy status, indicating that the late phase of the TCA cycle rather than the first phase is essential for fumarate elevation. In addition, the energy substrates responded to gluconeogenesis other than ketone bodies, fatty acids, amino acids, and carbohydrates, and they did not share intermediate conversion processes of the TCA cycle in the course of entry into this cycle (Dedkova & Blatter, 2014). Thus, herein, the elevation of the Nrf2 activator, fumarate, may be due to the unique characteristics of ketone metabolism, namely the activation of TCA cycle flux by the upregulation of a concomitant process of TCA cycle intermediate conversion and ketolysis in response to prominent sustained supplementation of 3HB in an energy shortage.

During glucose depletion, 3HB is used as an alternative source of energy. Although to a relatively smaller extent compared to hepatic ketogenesis, astrocytes possess the ability to generate 3HB (Thevenet et al., 2016), presumably to supply fuel for neurons. In the 3HB‐s and SF groups, single elevation of serum and vitreous 3HB did not prevent retinal degeneration and decrease in PPP metabolites after ONVT. In addition, ONVT cut off the oxygen supply through the arterial blood that is essential to generate energy by using 3HB through mitochondrial oxidative phosphorylation. Besides results showing increased nuclear levels of Nrf2 before ONVT due to 3HB elevation, it can be assumed that 3HB may not act as a significant source of energy, but as a signaling intermediate that induces multiple antioxidant actions to inhibit retinal degeneration induced by ONVT.

Despite providing novel insights, this study presents some limitations. Our results were limited to the short‐term effects of CR in acute‐phase retinal degeneration using young and middle‐aged rats. Further studies investigating the effect of extended CR on chronic retinal degeneration with aging will provide insights into the role of the association between 3HB, fumarate, and Nrf2 in aging‐related retinal diseases.

**Figure 6:**
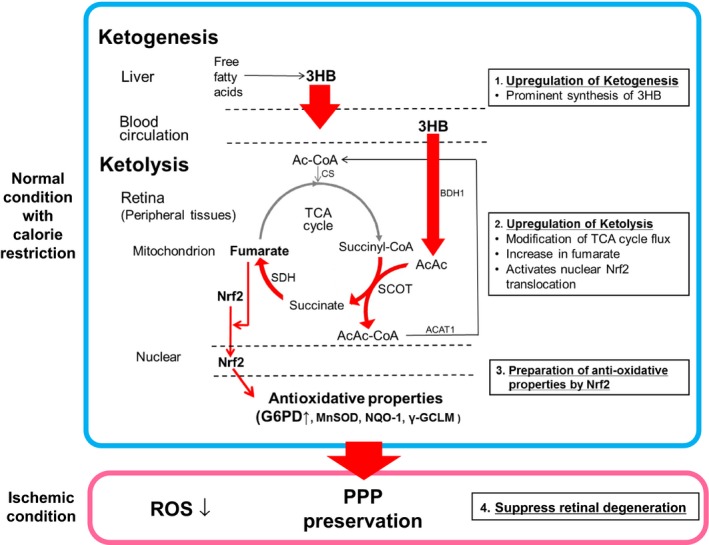
The hypothesized protective effect of CR on retinal degeneration by ONVT: the pivotal role of elevated endogenous 3HB via ketolysis. CR protects the retina from degeneration as follows: (1) CR upregulates ketogenesis in the liver, (2) a prominent increase is observed in the blood and retinal 3HB concentration during CR, (3) adaptive upregulation of the ketolytic pathway through its rate‐limiting enzyme, SCOT, in the retina, (4) acceleration of the TCA cycle flux with an increase in fumarate, an Nrf2 activator and TCA cycle intermediate, (5) translocation of Nrf2 upregulates its downstream antioxidative factors; G6PD, a rate‐limiting enzyme of antioxidative PPP, MnSOD, NQO‐1, and ɤ‐GCLM, and (6) CR suppresses retinal degeneration through preservation of PPP and antioxidative enzymes during ischemic conditions after ONVT. 3HB: 3‐hydroxybutyrate. AcAc: acetoacetate. AcAc‐CoA: acetoacetyl‐CoA. Ac‐CoA: acetyl‐CoA. SCOT: succinyl‐CoA‐3‐oxaloacid CoA transferase. SDH: succinate dehydrogenase. BDH1: D‐3‐hydroxybutyrate dehydrogenase. ACAT1: acetyl‐CoA acetyltransferase 1. CS: citrate synthase. PPP: pentose phosphate pathway

In the present study, we propose a novel mechanism for the antioxidative effect of endogenous 3HB during CR (Figure 6). Calorie restriction‐initiated ketogenesis, a prominent 3HB synthesis pathway in hepatic tissue, increases the 3HB concentration in blood and peripheral tissues. This increase adaptively upregulates the ketolytic pathway through its rate‐limiting enzyme SCOT, and is accompanied by an acceleration of the TCA cycle flux with an increase in fumarate, an antioxidative Nrf2 activator and TCA cycle intermediate. Nuclear translocation of Nrf2 upregulates its various downstream antioxidative factors.

Ketolysis is a well‐recognized cellular metabolic defense mechanism against energy deprivation, which is fundamental to peripheral tissues. Nrf2 is ubiquitously expressed in important cell defense and survival pathways under various physiological and pathological conditions. Therefore, it can be assumed that the mechanism underlying the antioxidant effect of 3HB participates in not only retinal protection, but also the ubiquitous, wide‐ranging health benefits of CR. Investigations of dietary intervention that focus on alterations in ketone body metabolism, especially on the 3HB‐utilizing process, ketolysis, may be a straightforward CR mimic strategy for various aging‐associated disorders, including retinal degeneration with vision loss such as glaucoma.

## 4 EXPERIMENTAL PROCEDURES

### 4.1 Animals

Seven‐ and 50‐week‐old male Wister rats were purchased from Charles River Laboratories Japan Inc. (Yokohama, Japan). Their body weight was approximately 200 g and 700–800 g, respectively. All animal experiments were performed under the following conditions: room temperature, 23 ± 2°C; relative humidity, 60% ± 10%; and an alternating 12‐hr light–dark cycle (8 a.m. to 8 p.m.). Animals were acclimatized before the experiments for at least a 1‐week period with food (MF; Oriental Yeast, Tokyo, Japan) and water available ad libitum (AL). Following the experiment, all animals were euthanized with an overdose of pentobarbital sodium (120 mg/kg). All animals were used according to the Association of Research and Vision in Ophthalmology (ARVO) statement for the Use of Animals in Ophthalmic and Vision Research. The protocol for this study was approved by the Ethics Committee on Animal Research of the Keio University School of Medicine (Approval No. 12002).

### 4.2 Experimental design

The following five groups were created: ad libitum (AL), provided access to food every other day (intermittent fasting, IF), 24‐hr fasting (single fasting, SF), 7 days of twice‐daily subcutaneous injections of 3HB with ad libitum (3HB‐r), and single‐dose subcutaneous injection of 3HB with ad libitum (3HB‐s). Body weight was recorded daily.

### 4.3 Calorie intake

Food intake was recorded daily (AL and 3HB‐r) or every other day (IF) during the experiment. Total calorie intake was calculated as the sum of food‐derived calorie intake with (3HB‐r) or without (AL and IF) exogenously injected 3HB‐derived calorie intake —given calorie intake values of 3.59 kcal/g derived from food and 4.96 kcal/g from 3HB.

### 4.4 Calorie restriction

For IF, rats were provided with unlimited access to food every other day for 7 days. Animals were used for experiments 24 hr after the last fasting started. For SF, after the acclimation period, AL treatment was continued for 6 days. Subsequently, rats had only access to water for 24 hr and were then used for experiments.

### 4.5 3HB administration

Sodium D‐3‐hydroxybutyrate (3HB, Ophtecs Corporation, Ltd., Hyogo, Japan) was used. It was dissolved in saline and injected into the dorsal skin of rats at 1,000 mg/kg.

### 4.6 Optic nerve and central retinal blood vessel transection

The rats were systemically anesthetized with pentobarbital (64.8 mg/kg) and the ocular surface was locally anesthetized with benoxil eye drops (Santen Pharmaceutical, Osaka, Japan). Then, the dorsal conjunctiva was incised and the intraorbital optic nerve with the retinal central artery and vein was transected. Sham operation involved conjunctival incision only. One, 4, or 8 hr after transection, rats were euthanized by exsanguination through cutting the carotid artery, after being deeply anesthetized with sodium pentobarbital (120 mg/kg, i.p.). The retinas were then isolated.

### 4.7 Pathological evaluation of retinal degeneration

After euthanization, eyeballs were immediately excised and then fixed in Davidson's solution (McKay, Steele, Ahmed, Johnson, & Ratcliffe, 2009) for at least 48 hr.

For hematoxylin and eosin (H&E) staining, samples were dehydrated with ethanol and xylene and then embedded in paraffin. A series of 4‐μm sections was obtained with a microtome. Sections were deparaffinized with xylene, ethanol, and water, and then routine H&E staining was performed. Morphometric analysis of retinal thickness was performed to quantitatively analyze retinal edema degeneration (Liu et al., 2016). Three areas of retina H&E‐stained sections were photographed per animal (magnification ×4, 600 μm in length). The average of retinal thickness from GCL to RPE in each photograph was measured by BZ‐II Analyzer software (Keyence, Osaka, Japan). All sections were analyzed in a blinded manner.

For transmission electron microscopic observation, paraffin‐embedded retinas were re‐embedded in epoxy resin by the method previously reported (Widéhn & Kindblom, 1988). Briefly, retinal tissues in the paraffin blocks were deparaffinized and stained in a 0.01% toluidine blue/absolute ethanol solution, infiltrated in a propylene oxide, and embedded in epoxy resin. The ultrathin sections were made using a diamond knife, stained with uranyl acetate and lead citrate, and examined using an electron microscope (JEM 1400Plus; JEOL Ltd., Tokyo, Japan).

### 4.8 Retinal viability

Freshly isolated neural retinas were immersed in a 96‐well culture plate containing 100 μl of serum‐free neurobasal medium and 10 μl of WST‐8 (Cell Counting Kit‐8, Dojindo, Kumamoto, Japan) per well, and then incubated (37°C, 5% CO2) for 1 hr immediately after euthanization. After incubation, the optical density of the culture supernatant was measured at 450 nm using a microplate reader (SpectraMax Paradigm; Molecular Devices, Sunnyvale, CA, USA).

### 4.9 Metabolomic analysis

A total of 10 groups, AL, SF, IF, 3HB‐r, and 3HB‐s with or without transection, underwent analysis. Retinas harvested 1 hr after ONVT were used. Two retinas were pooled for each measurement. Analyses of a total of 109 cations and 91 anions by capillary electrophoresis–mass spectrometry were carried out as previously described (Soga & Heiger, 2000; Soga et al., 2009, 2003) with some modifications. *Z*‐scored averages of three samples (*n* = 3) were used for the construction of a clustered heatmap using R software (http://www.r-project.org/) with the function “heatmap.2”.

### 4.10 Measurement of reactive oxygen species (ROS) generation in retina

Reactive oxygen species generation from freshly isolated retinas were measured 30 min after ONVT using the ROS‐sensitive fluorescence indicator 2′,7′‐dichlorofluorescein diacetate (DCFH‐DA, Molecular Probes, Eugene, OR, USA). After euthanization, their eyeballs were immediately excised and the retina was isolated in ice‐cold phosphate‐buffered saline (PBS). Excised retinas were homogenized in PBS (25 mg tissue weight/ml) using a zirconia ball mill (AS ONE corporation, Osaka, Japan). Retinal homogenates were incubated with DCFH‐DA (final concentration, 30 μm) for 1 hr at 37°C. Then, homogenates were washed three times with PBS by centrifugation at 1,200 g for 3 min at 4°C. Finally, washed homogenate were resuspended in PBS, and analyzed at an excitation wavelength of 480 nm and emission wavelength of 530 nm, using a Synergy 4 plate reader (BioTek Company, Winooski, VT, USA). ROS generation levels were calculated as the ratio of the sham‐operated eye.

### 4.11 Detection of oxidative stress in the retina by Nε‐(hexanoyl) lysine immunostaining

After euthanization, rats’ eyeballs were harvested and fixed in 10% formalin solution (Wako, Osaka, Japan) for at least 48 hr. Samples were embedded in OCT compound (Miles Laboratories, Elkhart, IN, USA) and frozen in liquid nitrogen. A series of 4‐μm sections was obtained with a microtome. Sections were incubated with 5% BSA and 0.25% Triton X‐100 in PBS for 30 min and then incubated overnight with a primary antibody solution. Anti‐HEL antibody (JaICA, Shizuoka, Japan) at 2 μg/ml was used as the primary antibody. After incubation with the primary antibodies, Alexa Fluor^®^‐conjugated antibody (Life Technologies, Carlsbad, CA, USA) was applied. For nuclear staining, Hoechst33342 (Dojindo, Kumamoto, Japan) was used with the secondary antibody. Sections were mounted and examined with a confocal fluorescence microscope (LSM 710, Carl Zeiss, Oberkochen, Germany).

### 4.12 Western blot analysis

After euthanization, eyeballs were harvested and isolated retinas were homogenized with RIPA buffer (50 mm Tris/HCl pH 7.5, 150 mm NaCl, 1.0% Igepal CA‐630) containing protease inhibitor cocktail (Complete Mini; Roche Diagnostics, Basel, Switzerland), and 10 μm MG132 (Peptides International, Louisville, KY, USA). Samples were centrifuged at 4°C, 20,400 g, for 5 min, and the protein concentration was determined by the DC protein assay kit (Bio‐Rad, Hercules, CA, USA). Then, the same volume of 2× Laemmli sample buffer was added and 5% β‐mercaptoethanol. After boiling, the sample was separated by SDS‐PAGE. After electrophoresis, the proteins were transferred onto a PVDF membrane. The membranes were incubated with 5% skim milk or 5% BSA + 0.1% Tween‐20 in TBS, and then with primary antibodies. The primary antibodies were as follows: anti‐G6PD antibody (Atlas antibodies AB, Stockholm, Sweden) at 1:200, anti‐MnSOD antibody (Santa Cruz Biotechnology, Dallas, TX, USA) at 1:200, anti‐NQO‐1 antibody (Cell Signaling Technology, Danvers, MA, USA) at 1:1,000, anti‐ɤGCLM antibody (Santa Cruz Biotechnology) at 1:200, and anti‐β‐actin antibody (Sigma‐Aldrich, St. Louis, MO, USA) at 1:2,000, anti‐BDH1 antibody (Proteintech, IL, USA) at 1:1,000, anti‐SCOT antibody (Abnova, Taipei, Taiwan) at 1:500, anti‐ACAT1 antibody (Proteintech, IL, USA) at 1:1,000, anti‐SDHA antibody (Cell Signaling Technology) at 1:1,000, and anti‐CS antibody (Cell Signaling Technology) at 1:1,000. After incubation with the primary antibodies, an alkaline phosphatase‐conjugated antibody (Promega, Madison, WI, USA) was applied. NBT/BCIP was used as a colorimetric substrate. The images of the membranes were acquired with a scanner (GS‐800 Calibrated Densitometer; Bio‐Rad). Intensities of the bands were quantitated using ImageJ software (National Institutes of Health, Bethesda, MD, USA). The ratio of the band intensity to the β‐actin was calculated.

### 4.13 Translocation of nuclear Nrf2 in the retina

For the Western blot analysis, retinal nuclear protein was extracted from freshly isolated retina using NE‐PER Nuclear and Cytoplasmic Extraction Reagents (Pierce Biotechnology, Rockford, IL, USA) according to the manufacturer's instructions. The extracted retinal nuclear proteins were applied to Western blotting analysis as described above. Anti‐Nrf2 antibody (MBL, Nagoya, Japan) at 1:1,000 and anti‐TBP antibody (Cell Signaling Technology) at 1:1,000 were used as primary antibodies. The ratio of the band intensity of Nrf2 to that of TBP was calculated.

For the localization analysis by immunohistochemical staining, rats were euthanized with an overdose of pentobarbital sodium (120 mg/kg) prior to ONVT. The above‐mentioned immunostaining protocol was applied as Nrf2 staining method. Anti‐Nrf2 antibody (Abcam, Cambridge, UK) was used as the primary antibody at 1:100. The colocalization of Hoechst‐stained nuclei and immunostained Nrf2 was observed by using a confocal fluorescence microscope (LSM 710).

### 4.14 Measurement of glucose‐6‐phosphate dehydrogenase activity

Total glucose‐6‐phosphate dehydrogenase (G6PD) activity levels per retinal weight were measured using the Glucose‐6‐Phosphate Dehydrogenase Assay Kit (Sigma‐Aldrich) according to the manufacturer's instructions.

### 4.15 Measurement of fumarate

The total fumarate content per retina was measured using the Fumarate Assay Kit (Sigma‐Aldrich) according to the manufacturer's instructions.

### 4.16 Measurement of retinal oxygen consumption rate

Immediately after euthanization, their eyeballs were immediately excised and the retina was isolated in ice‐cold saline. The retina was spread flat, and hollowed out with 1.5‐mm biopsy punch (Kai Industries, Gifu, Japan) and used for measurement of retinal basal OCR. Basal OCR was measured by XF 24‐3 flux analyzer (Seahorse Biosciences, Billerica, MA, USA). The retinal basal OCR was measured in 0, 5, and 10 mm 3HB in saline for 10 min, followed by saline for 20 min as the baseline. All measurements were performed at 37°C. The percentage to the baseline value of each 3HB concentration was calculated.

### 4.17 Nrf2‐deficient mouse

Seven‐week‐old male C57BL6J Nrf2 (−/−) knockout mice were used (Banning, Deubel, Kluth, Zhou, & Brigelius‐Flohe, 2005). Control mice were age‐matched male C57BL/6 WT mice purchased from Charles River Laboratories Japan Inc. (Yokohama, Japan).

All experimental protocols, including calorie restriction, 3HB administration, and ONVT, were performed following the protocol used for the rat study.

### 4.18 Statistical analysis

We used Student's *t*‐test for comparison of the two groups and the Dunnett's test for multiple comparisons. Differences between measured variables were considered significant if the resultant *p*‐value was .05 or less. Analysis was performed using JMP version 12.0.1 (SAS Institute, Cary, NC, USA).

## CONFLICT OF INTEREST

None declared.

## AUTHOR CONTRIBUTIONS

S.N., K.T., and Y.I. designed the research plan; Y.I., T.I., R.H, E.O, and E.I., performed the experiments; Y.I. analyzed the data and wrote the original manuscript; S.N. performed substantial editing of the manuscript; M.I. performed transmission electron microscopic analysis; T.S. performed metabolomic analysis. All authors participated sufficiently in this work.
