# Supplementary material for: Ketone body 3‐hydroxybutyrate mimics calorie restriction via the Nrf2 activator, fumarate, in the retina
Source: Aging Cell. 2017 Nov 9;17(1):e12699. doi: 10.1111/acel.12699 (PMC5770878; doi:10.1111/acel.12699)
Supplement: Supplementary file 1 [file ACEL-17-na-s001.pptx]

## Slide 1
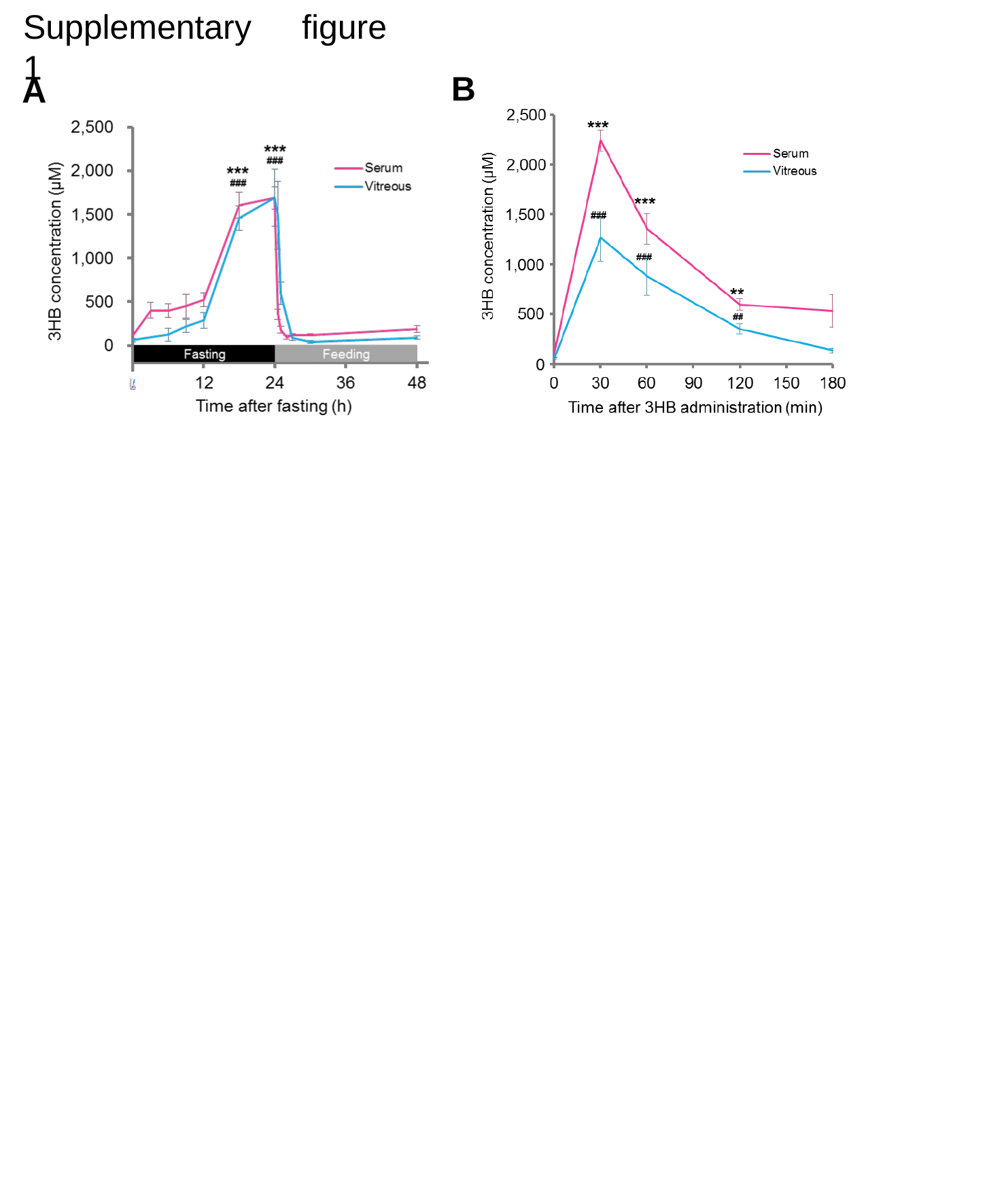

Supplementary　figure 1
B
A

## Slide 2
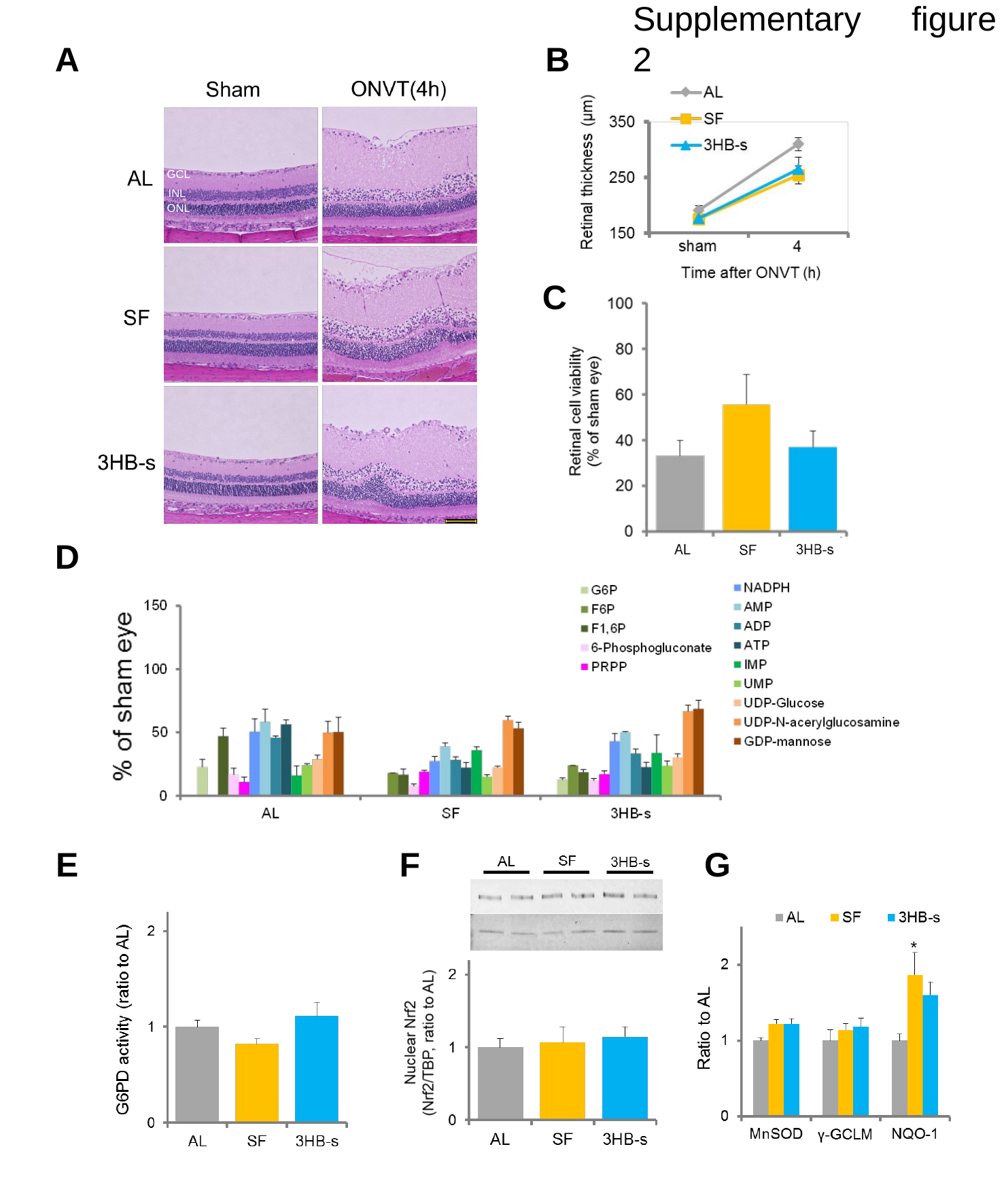

Supplementary　figure 2
A
B
GCL
INL
ONL
C
D
E
F
G
*

## Slide 3
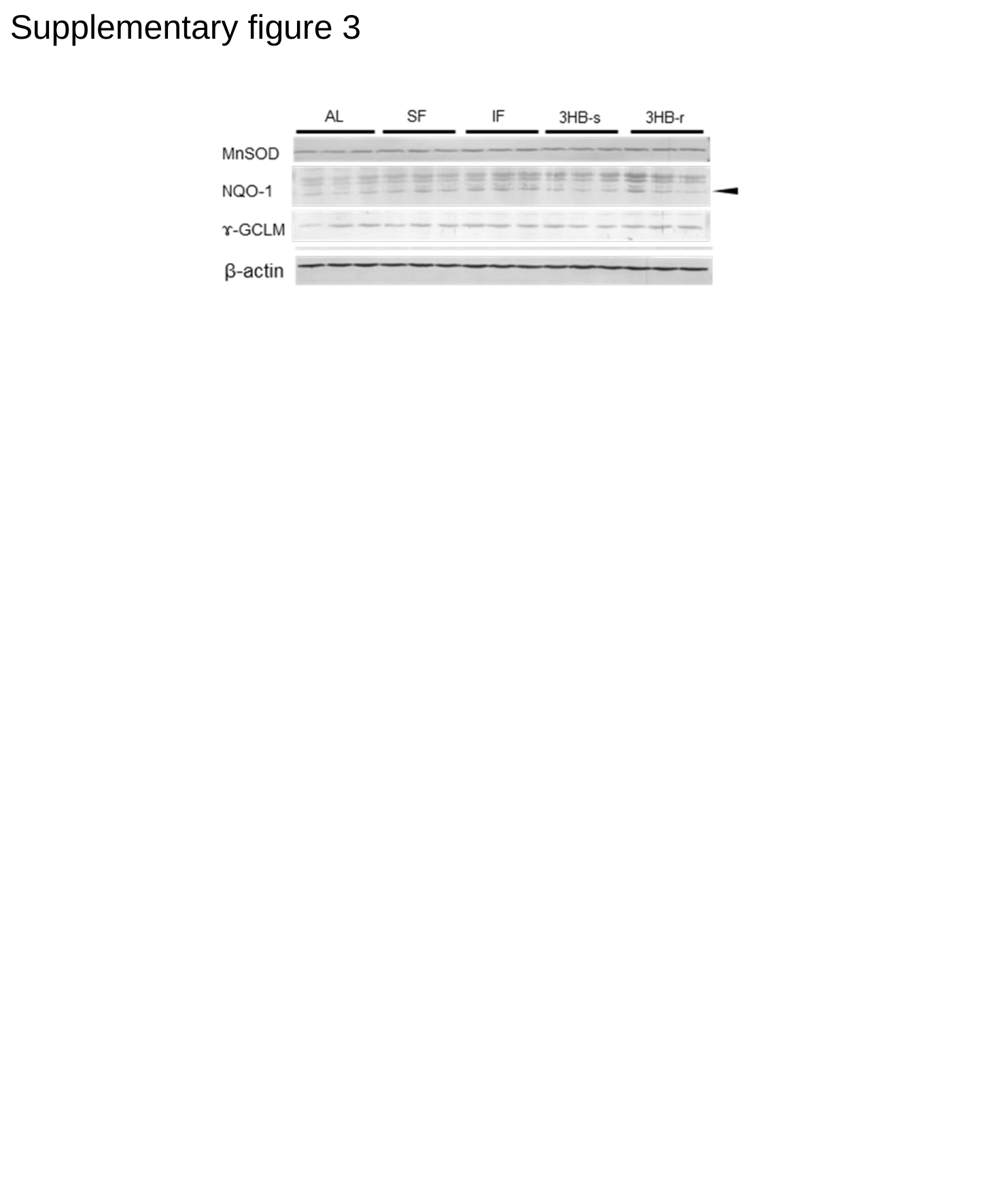

Supplementary figure 3

## Slide 4
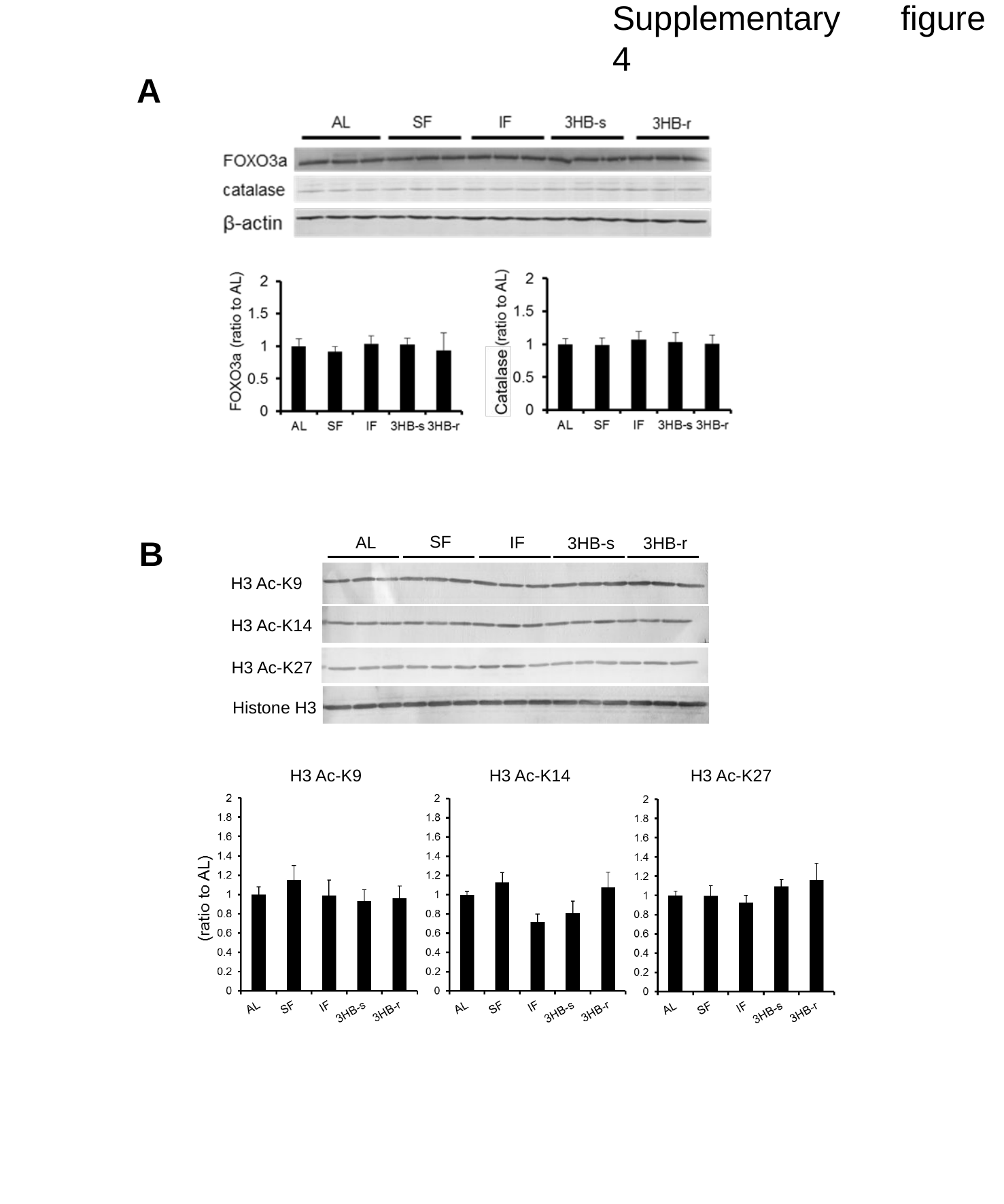

Supplementary 　figure 4
A
SF
AL
IF
B
3HB-s
3HB-r
H3 Ac-K9
H3 Ac-K14
H3 Ac-K27
Histone H3
H3 Ac-K9
H3 Ac-K27
H3 Ac-K14
