# Supplementary material for: Ketone body 3‐hydroxybutyrate mimics calorie restriction via the Nrf2 activator, fumarate, in the retina
Source: Aging Cell. 2017 Nov 9;17(1):e12699. doi: 10.1111/acel.12699 (PMC5770878; doi:10.1111/acel.12699)
Supplement: Supplementary file 2 [file ACEL-17-na-s002.docx]

**Supplementary Table 1. Biochemical analysis of rat serum.**

|  | AL | IF | 3HB-r |
| --- | --- | --- | --- |
| GLU (mg/dL) | 130.43 ± 7.32 | 64.33 ± 6.48^***^ | 129.33 ± 7.05 |
| TL (mg/dL) | 359.00 ± 14.14 | 212.67 ± 6.34^***^ | 299.58 ± 7.07^**^ |
| TG (mg/dL) | 122.71 ± 10.20 | 28.83 ± 2.60^***^ | 86.67 ± 6.23^**^ |
| NEFA (µEq/L) | 212.00 ± 13.66 | 356.50 ± 40.68^**^ | 159.17 ± 15.05 |

Mean ± SE, n = 6–7, **P* < 0.05 ***P* < 0.01 ****P* < 0.001 versus AL.

Serum were collected at 4 hours after fasting and 30 minutes after 3HB administration for IF and 3HB-r, respectively.

**Supplementary Table 2. Total food intake.**

|  | AL | IF | 3HB-r |
| --- | --- | --- | --- |
| Total food intake(g) | 156.78 ± 2.31 | 92.96 ± 2.50^***^ | 147.50 ± 2.09^*^ |

Mean ± SE, n = 5, **P* < 0.05, ****P* < 0.001 versus AL.
