# Supplementary material for: Ketone body 3‐hydroxybutyrate mimics calorie restriction via the Nrf2 activator, fumarate, in the retina
Source: Aging Cell. 2017 Nov 9;17(1):e12699. doi: 10.1111/acel.12699 (PMC5770878; doi:10.1111/acel.12699)
Supplement: Supplementary file 3 [file ACEL-17-na-s003.docx]

# **Supplementary information**

## **Supplementary Materials and Methods**

## **Measurement of 3-hydroxybutyrate concentration in serum and vitreous**

Rat blood was collected from abdominal aorta under deep anesthetized with intraperitoneal sodium pentobarbital (64.8 mg/kg). Serum was separated from blood by centrifuged for 20 minutes at 3000 rpm, 25°C. Vitreous fluid was collected from incision of the sclera of the freshly- excised eyeball. Concentrations of 3HB in collected serum and vitreous were measured by using 3HB KAINOS (KAINOS Laboratories, Tokyo, Japan) according to the manufacturer’s instructions.

**Blood biochemistry**

Serum biochemical parameters were analyzed at last treatment day (day 7) Serum were collected at 24 hours after fasting and 30 minutes after 3HB administration for IF and 3HBr, respectively. Triglycerides (TG), phospholipids (PL), total cholesterol (T-CHO), non-esterified fatty acids (NEFA), glucose (GLU) and total lipids (TL) levels were measured at the Nagahama Life-science Laboratory of Oriental East (Shiga, Japan).

## **Extraction of the total histone fraction and western blotting**

Extraction of the total histone fraction from the retina was performed by using the EpiQuik Total Histone Extraction Kit (Epigentek, New York, USA) according to the manufacturer’s instructions. The obtained fraction was used for western blotting as above. Primary antibodies were as follows: anti-HistoneH3 (Cell Signaling Technology) at 1:2,000, anti-acetyl-HistoneH3 (Lys9) (Cell Signaling Technology) at 1:1,000, anti-acetyl-HistoneH3 (Lys14) (Cell Signaling Technology) at 1:500, anti-acetyl-HistoneH3 (Lys27) (Cell Signaling Technology) at 1:1,000.


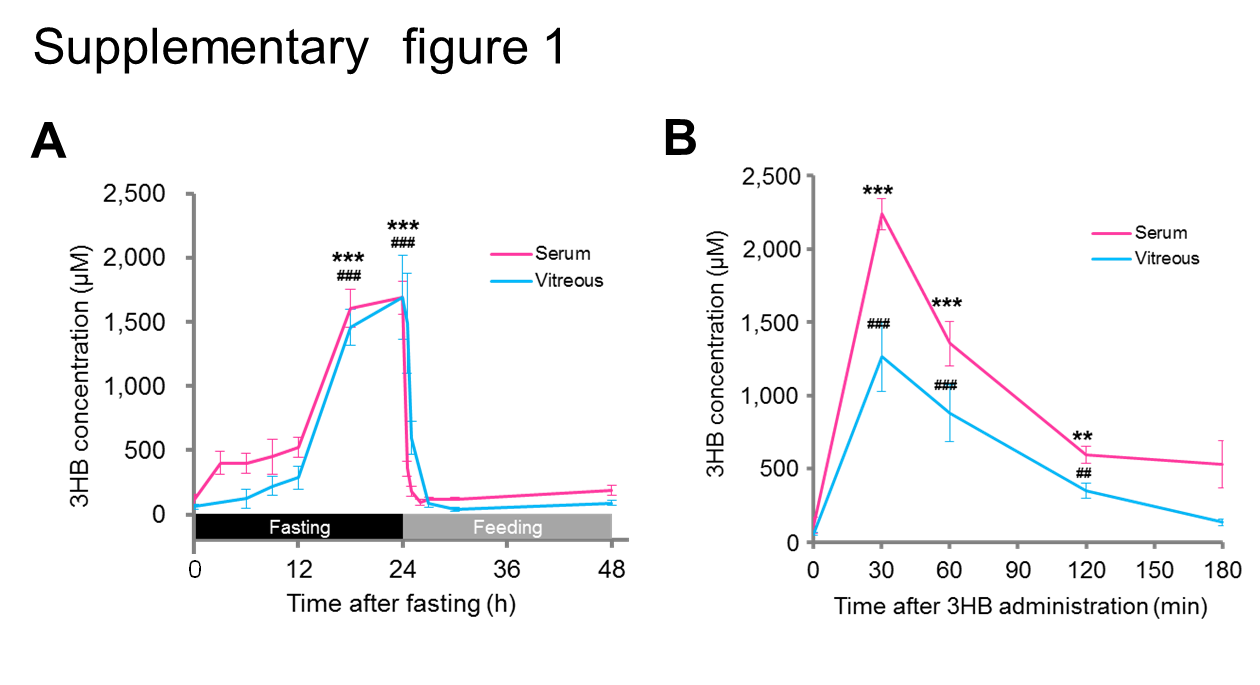


**Figure S1. Changes in 3HB concentration of serum/vitreous during fasting and 3HB subcutaneous administration.**

(A) Change in 3HB concentration of serum/vitreous during fasting and subsequent feeding. (B) Kinetics of 3HB concentration of serum/vitreous after 3HB subcutaneous administration (1000 mg/kg). All data represent the mean ± SE (n = 4–6), ****P* < 0.001 versus 0-min serum, ##*P* < 0.01, ###*P* < 0.001 versus 0-min vitreous (A, B).


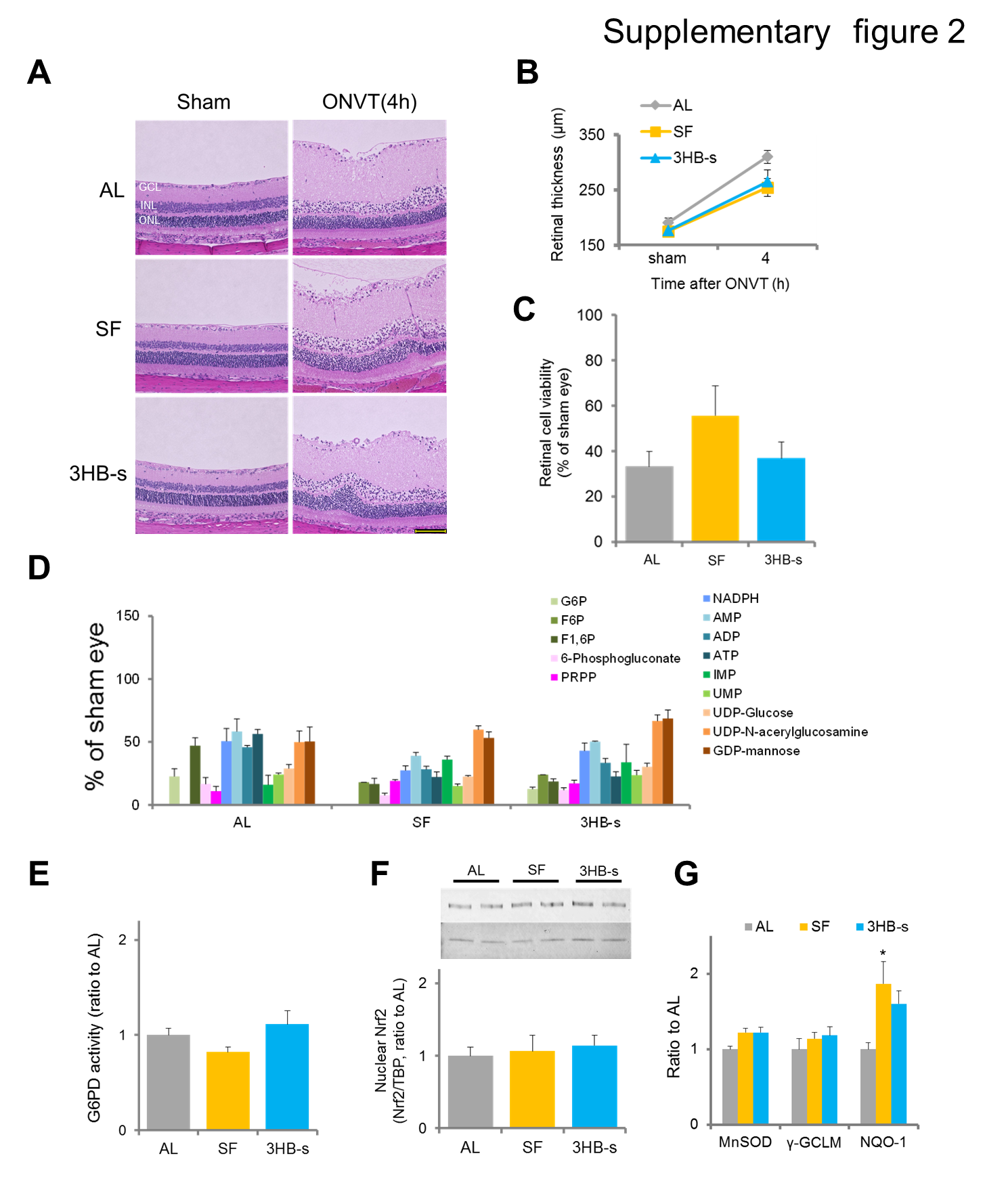


**Figure S2. Single fasting and 3HB administration did not prevent retinal degeneration or preserve PPP after ONVT and activated Nrf2 pathways prior to ONVT.**

(A) Retinal viability (n = 6). (B) Histopathological change of the retina (H&E staining. Scale bar, 100 µm) (C) Morphometric analysis of retinal degeneration. Analysis was performed at 4 hours after the ONVT (n = 6). (D) Changes in PPP and related metabolites after ONVT (n = 3). (E) Enzyme activity of G6PD (n = 5–6). (F) Nuclear accumulation level of Nrf2 (n = 5). TBP is loading control. (G) Expression levels of the downstream factor of Nrf2, MnSOD (left), NQO1 (middle), and ɤ-GCLM (right). Images used for quantification are shown in Supplemental Figure S3A. All data represent the mean ± SE. **P* < 0.001 versus AL.


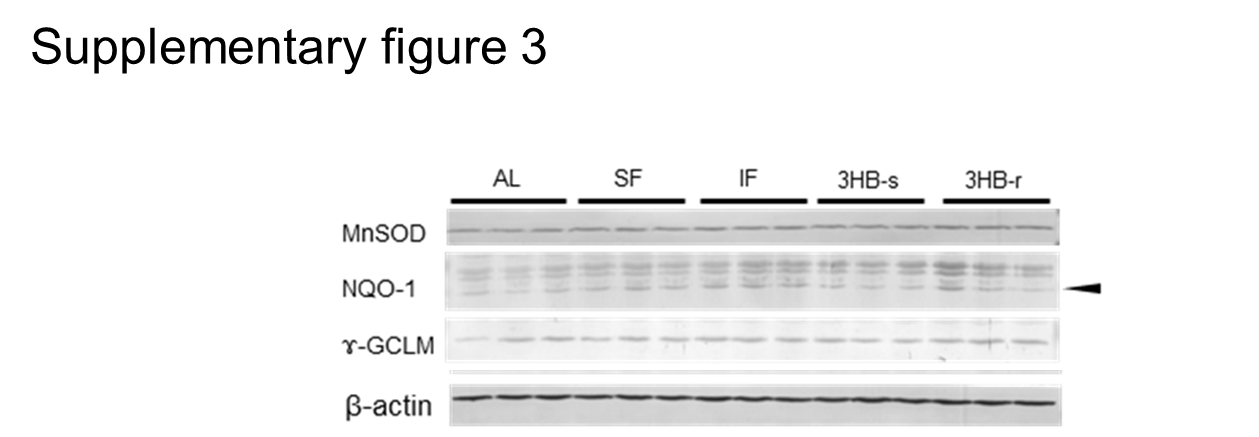


**Figure S3. Western blotting images of the downstream factors of Nrf2 and other oxidative stress related proteins prior to ONVT.** The quantitative data are shown in figures 3 and S2. Western blotting images of the downstream factor of Nrf2, MnSOD, NQO1, and ɤ-GCLM.


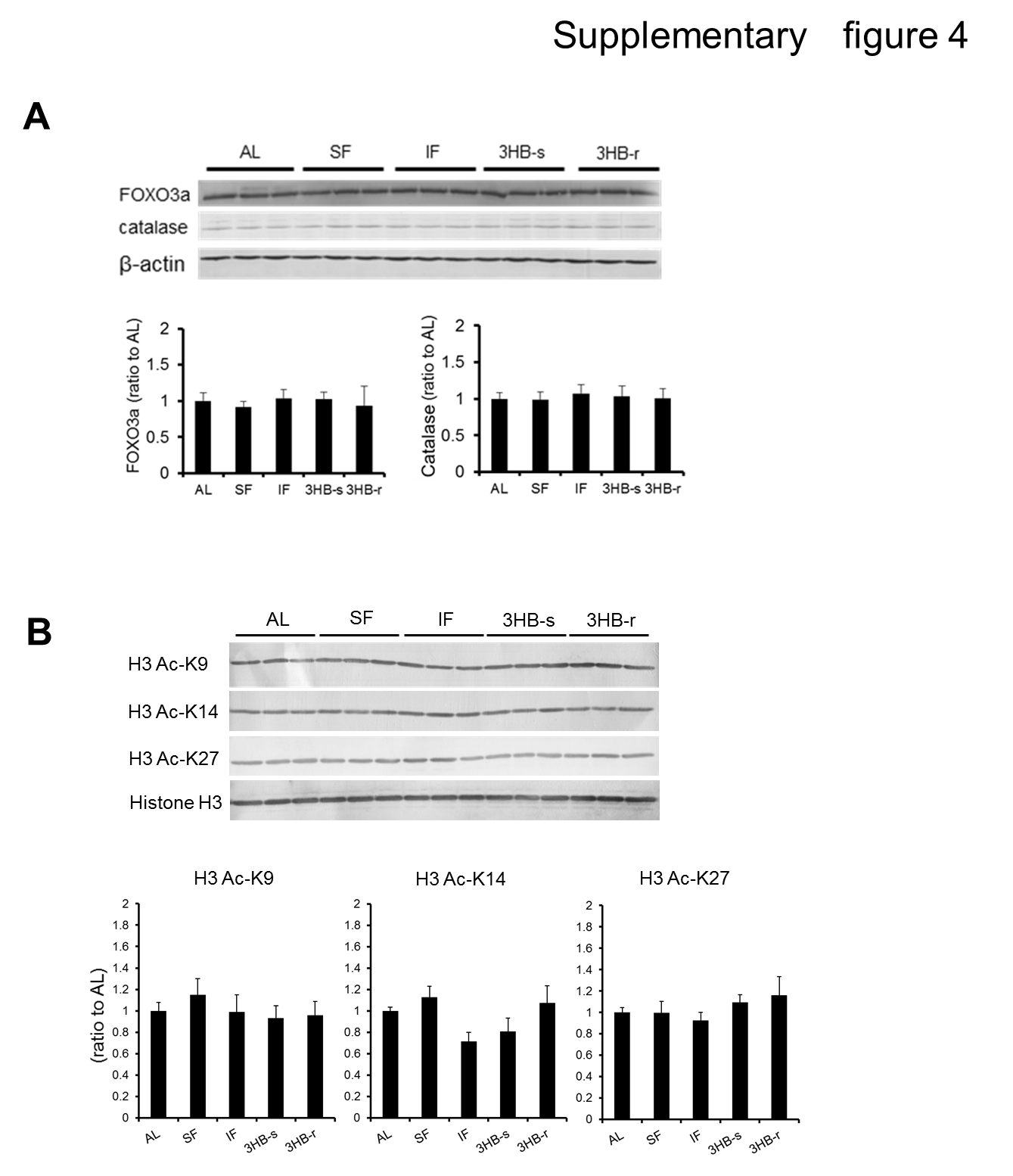


**Figure S4. Western blotting images of the acetylation levels of histone H3 and downstream antioxidative factors prior to ONVT.**

(A) Western blotting images of histone H3 K9, K14, and K27. The graph indicates the ratio of the band intensity to the Histone H3 (n = 5). (B) Western blotting images of FOXO3a and catalase. The graph indicates the ratio of the band intensity to the β-actin. All data represent the mean ± SE, n = 5.
